# The role of residents in medical students’ neurology education: current status and future perspectives

**DOI:** 10.1186/s12909-020-02036-1

**Published:** 2020-04-16

**Authors:** Zafer Keser, Yvo A. Rodriguez, Jennifer Tremont, Peggy H. Hsieh, Louise D. McCullough, Stefano Sandrone, Erin F. Stimming

**Affiliations:** 1 Neurology Department, UTHealth McGovern Medical School, 6431 Fannin Street, Suite 7.044, Houston, TX 77030 USA; 2 Internal Medicine Department, UTHealth McGovern Medical School, Houston, TX 77030 USA; 3 Department of Brain Sciences, Imperial College London, London, UK

**Keywords:** Neurology education, Clerkship, Medical students, Near-peer teaching

## Abstract

**Background:**

Neurophobia, a well-described fear of neurology, affects medical students worldwide and may be one of the factors contributing to a shortage of neurologists in the United States. Residents spend a considerable amount of time with medical students; therefore, we sought to understand better the impact neurology residents have on medical students during their neurology clerkship and their subsequent interest in neurology. We aimed to identify and implement strategies to decrease neurophobia and increase the number of students pursuing neurology as a career.

**Methods:**

Third-year medical students (*n* = 234) of UTHealth’s McGovern Medical School rotating through their neurology core clerkship completed two surveys regarding their rotation experiences. Surveys were completed anonymously before and after the clerkship to measure their interest and confidence in neurology and the impact of their interactions with the neurology residents during the clerkship. In parallel, residents participated in a teaching workshop focused on small group teaching to improve their teaching effectiveness. Non-parametrical comparison and ordinal regression analyses were utilized for data analyses.

**Results:**

Medical students reported a statistically significant increase in their confidence in managing neurological conditions and interest in pursuing a neurology residency after their clerkship. There was a significant association between the medical students’ overall rotation experience and the residents’ teaching effectiveness. The overall clerkship experience correlated with the medical students’ interest and confidence in neurology. There was a trend towards an increase in residents’ teaching effectiveness and students’ rotation experience after a resident teaching workshop. Additionally, of note, students who rotated on both and outpatient and inpatient sites during their clerkship reported an increased interest in neurology.

**Conclusion:**

Our study supports that resident-led teaching efforts are important in improving medical students’ neurologic education and their interest in neurology**.** Our data also supports that the interest in neurology increased for medical students after their neurology clerkship. We examined future strategies to implement “near-peer” teaching activities to enhance the medical students’ neurologic educational experience. These strategies could potentially mitigate neurophobia and ultimately lead to a much-needed increase in future neurologists.

## Background

Despite the high lifetime risk of disabling and fatal neurological conditions [1] and the increasing aging population [2], the number of medical students pursuing neurology remains low [3]; hence a shortage of neurologists of 19% is predicted by 2025 [4]. To counteract this shortage and increase interest in pursuing neurology as a career, it is essential to evaluate fundamental aspects of the neurological educational system.

One of the putative causes contributing to the diminished interest in neurology is ‘neurophobia,’ a term coined by Dr. Jozefowicz in 1994 [5]. Neurophobia is characterized as intimidation and boredom with the neurosciences as well as difficulty grasping the main concepts in neurology. The incidence of neurophobia among medical students has been reported to be as high as 50% [5]. Over the last 25 years, several strategies have been proposed to mitigate neurophobia, increase medical students’ interest in neurology [6, 7], and further increase the pursuit of neurology as a career path.

A recently proposed strategy is educating the educator, as resident-as-educator training might help with improving educational experiences for students [8]. A study from an obstetrics and gynecology clerkship revealed that a resident-driven mentoring program was beneficial for medical students’ career selection [9]. The importance of daily interactions between medical students and neurology residents has been hypothesized to be a vital component of the medical students’ education during their neurology rotation [10].

Residents play a significant role in teaching the basics of neurology during the third-year neurology clerkship, where they have direct interactions with students and, therefore, are at an opportune time to stimulate student interest in neurology. Although the neurology residents are anecdotally known to contribute to the medical students’ neurology education, the components of this contribution are not well characterized in the literature. In this study, we attempted to characterize the main determinants of the perceived impact of neurology residents on medical students’ attitudes towards neurology. In addition, the residents participated in a teaching workshop, after which we investigated the secondary effects on the medical students’ overall perceived experience.

## Methods

**Fig. 1:**
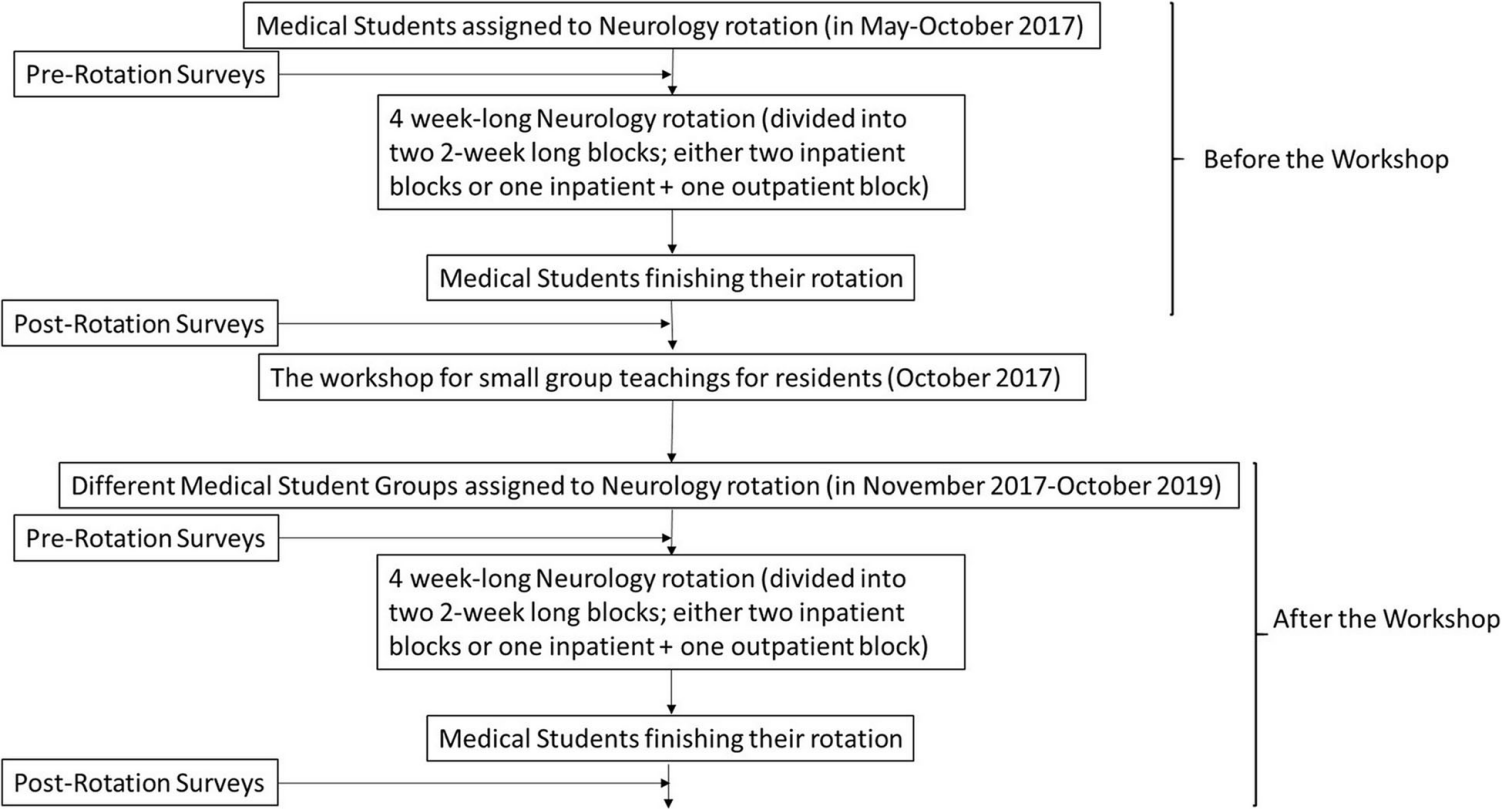
The diagram of the study overflow

Third-year McGovern Medical School (MMS) students rotating through their core four-week neurology clerkship between May 2017 and October 2019 were asked to participate in this study. Participants completed two anonymous online surveys regarding their perceptions of neurology and their overall experience during the clerkship. Surveys were completed before and immediately after completing the clerkship. The Qualtrics© online forum was used for the administration of the surveys (https://www.qualtrics.com/). The study overview is highlighted in Fig. 1.

The neurology residents underwent a one-time interactive hour-long workshop on small group teaching strategies led by a Ph.D. educator from the Office of Educational Programs at MMS. The objective of the workshop was to introduce effective teaching strategies and improve residents’ teaching skills. It included topics such as how learners process information, use of questioning skills, and the one-minute preceptor model. There was no assessment of residents’ teaching skills before and after the workshop.

Pre-rotation and post-rotation questionnaires included students’ level of interest in pursuing neurology as a career (scale: 1 = not interested at all, 2 = undecided, 3 = slightly interested, 4 = very interested) and confidence in managing neurological conditions (scale: 1 = not confident at all, 2 = not confident, 3 = somewhat confident, 4 = extremely confident). Post-rotation questionnaire also included students’ perception of the adequacy of time residents took to teach (scale: 1 = no teaching at all, 2 = not a lot of time spent on teaching, 3 = limited time, 4 = adequate time), the effectiveness of resident teaching (scale: 1 = extremely ineffective, 2 = somewhat ineffective, 3 = effective, 4 = very effective), the impact of residents on overall rotation experience (scale: 1 = very negative, 2 = negative, 3 = positive, 4 = very positive), overall value of the rotation (1 = not valuable at all, 2 = not valuable, 3 = somewhat valuable, 4 = very valuable) and residents’ bedside manner and professionalism (scale: 1 = definitely no, 2 = no, 3 = yes, 4 = definitely yes). See the Additional file 1 for the internally generated survey.

### Statistical analyses

Descriptive statistics and the Mann Whitney U test were used to analyze the change in the students’ interest in neurology along with their confidence level in neurology before and after the rotation. The pre−/post rotation data is collected from the same students, as were collected anonymously. As the data was gathered using Likert scales, ordinal regression analysis was used to determine the correlates of students’ overall experience during their clerkship, residents’ impact on their experience, their interest in neurology, and confidence in managing neurological conditions. We created four univariate regression models for the regression analyses. In the first model, the overall value of the clerkship was the dependent variable, and the time the residents spent teaching, the residents’ teaching effectiveness, and residents’ professionalism and bedside manner were independent variables. In the second model, the residents’ impact on the students’ neurology experience was treated as the dependent variable, and the time the residents spent teaching, the residents’ teaching effectiveness, and residents’ professionalism and bedside manner were independent variables. In the third and fourth models, the students’ interest in neurology and confidence in managing neurological conditions were the dependent variables respectively, and the time the residents spent teaching, the residents’ teaching effectiveness, residents’ professionalism, and bedside manner, residents’ impact on students’ neurology experience and students’ overall experience were the independent variables. The students rated residents’ teaching effectiveness before and after the teaching workshop. The residents’ teaching effectiveness and impact on students’ rotation experience was assessed by students who rotated through the neurology clerkship and interacted with the residents before the resident workshop (*n* = 58) and after the workshop (*n* = 176) using the Mann Whitney U Test. The medians of residents’ teaching effectiveness and their impact on students’ rotation experience rated by the students before and after the resident workshop were compared by the Mann Whitney U Test.

We also collected site-specific information from a subset of our students (*n* = 149). A group of students only rotated on inpatient sites (*n* = 50), and the other group rotated in both inpatient and outpatient sites (*n* = 99). We compared their overall experience of neurology, the perceived resident teaching effectiveness, the impact of residents’ teaching on students’ rotation experience, students’ interest, and perceived confidence in neurology. False discovery rate (FDR) of 10% was performed for the correction of multiple comparison analyses, and R statistical software (https://www.r-project.org/) was used for statistical analyses.

## Results

Three hundred seventy-one students were invited, of which 234 (63%) students completed the surveys. We did not collect demographic data from the students.

### Change of students’ interest and confidence in neurology after the rotation

**Fig. 2:**
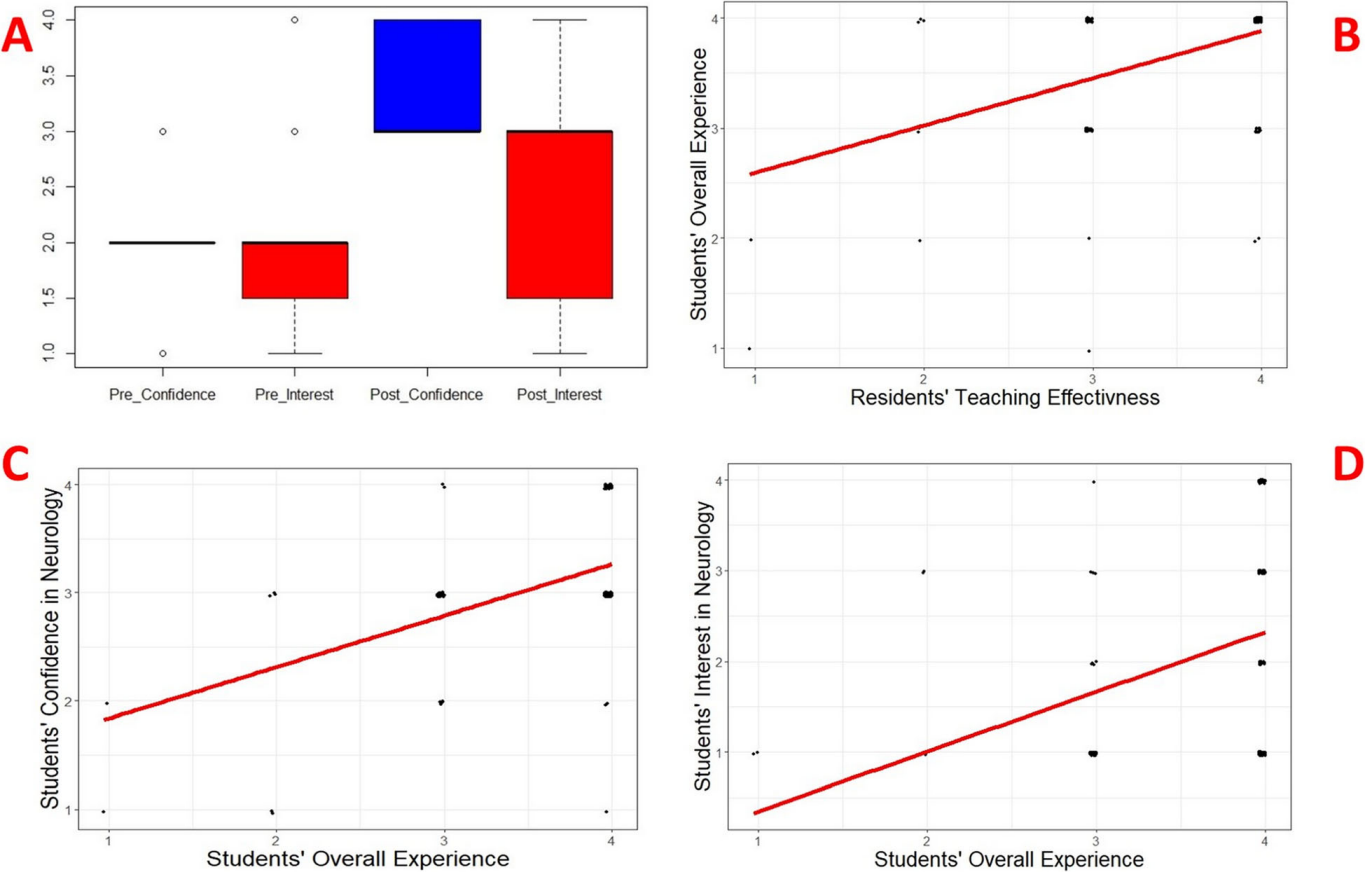
Change in students’ interest and confidence in neurology after the clerkship rotation and their correlates. Box-plot illustration of students’ confidence in managing neurological conditions and interest in pursuing neurology residency before and after neurology clerkship (**a**). Scatter-plot illustrations of the association between the overall value of the neurology rotation experience for medical students and the residents’ teaching effectiveness (**b**), students’ confidence in managing neurological conditions (**c**), and interest in pursuing neurology residency (**d**). Of note, minimal random noise added to scatter plots to illustrate number of data points better. Scales for confidence and interest are as follows; 1 = not interested/confident, 2 = undecided, 3 = somewhat confident/interested, 4 = extremely confident/interested. Scale for overall rotation value is as follows; 1 = not valuable at all, 2 = not valuable, 3 = somewhat valuable, 4 = very valuable. Scales for residents’ teaching effectiveness/residents’ impact on students’ rotation experience are respectively as follows; 1 = very ineffective/affected very negatively, 2 = ineffective/affected negatively, 3 = somewhat effective/affected positively, 4 = extremely effective/ affected very positively

Among the students who completed the surveys (*n* = 234), the median [interquantile range (IQR)] was 2 [0] in managing neurological conditions before the rotation, and this increased to 3[1] (*p* < 0.001) after the rotation (Fig. 2a). The median [interquantile range (IQR)] interest in pursuing neurology as a career increased from 2 [0.5] to 3 [1.5] (*p* = 0.001) upon completion of the rotation (Fig. 2a).

### Components of the residents’ effect on the students’ rotation experience

The ordinal regression analyses showed a significant association between the students’ overall rotation experience and resident teaching effectiveness (Fig. 2b and Table 1). Although the residents’ teaching effectiveness did not show a significant association with students’ interest in neurology and their confidence in managing neurological conditions (Fig. 2c and Table 1), their overall neurology clerkship experience was associated with interest (Fig. 2d and Table 1) and confidence in neurology (*p* < 0.001 and *p* < 0.001). Residents’ impact on the students’ rotation experience was significantly associated with residents’ teaching effectiveness, professionalism, and bedside manner, and time spent by residents to teach (Table 1).

**Table 1:**
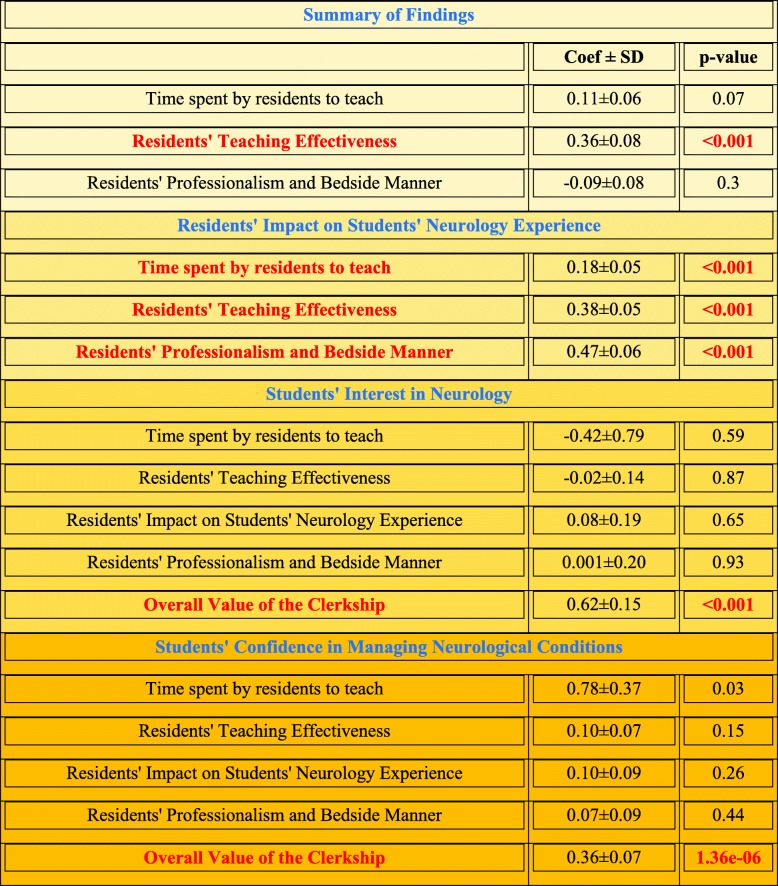
Summary of the results of ordinal regression analyses for the determinants of the overall value of the rotation, residents’ impact on the students’ clerkship experience, students’ confidence, and interest in neurology

Significant results are shown in red font

*Abbreviations*: *p-val p*-values, *SD* Standard deviation

Residents’ teaching effectiveness was significantly associated with improved overall clerkship experience of the students. Overall rotation experience was a positive predictor for students’ confidence and interest in neurology. Residents’ impact on medical students’ rotation experience was positively related to their perceived teaching efficiency and bedside manners as well as the amount of time they spent with the students.

### Effects of the resident workshop

Residents’ teaching effectiveness and their impact on students’ rotation experience showed a trend of increase after the workshop, but this did not reach statistical significance (*p* = 0.35).

### Effects of site-specificity

The additional outpatient experience did not lead to a significant difference in their overall experience (*p* = 0.75), their perceived confidence in managing neurological conditions (*p* = 0.39), the residents’ impact on their rotation experience (*p* = 0.9), residents’ perceived teaching effectiveness (*p* = 0.13). The students’ interest in neurology was higher in the group who rotated through an outpatient and inpatient site (median [IQR] 3[2] vs. 2[1], *p* = 0.03). This significance became insignificant after FDR correction.

## Discussion

Our results demonstrate that, not surprisingly, third-year medical students’ report a positive and statistically significant correlation with their interest and confidence in neurology after their neurology clerkship. The students also reported substantial benefits from the residents’ involvement and teaching skills during the clerkship. Our study supports that residents provide additional value for the neurologic education of medical students. While this is not unexpected, this has not previously been quantified and published.

Near-peer teaching has the advantage of providing social and cognitive congruence between the student and the teacher [10]. Our findings align well with previous studies showing that students benefit academically and professionally in a near-peer teaching setting [11, 12]. However, students taught by peers do not have different outcomes from those taught by faculty [13].

A training workshop on teaching strategies to the residents may potentially optimize medical students’ experience during their neurology rotation and increase their confidence in managing neurological conditions. Although there was a slight increase in students’ overall experience after the workshop, the difference did not reach statistical significance, possibly because the workshop was provided only once. We, therefore, believe that residents should be introduced to core educational concepts through formal online or in-person teaching workshops. Minimal investments in enhancing residents’ teaching ability can lead to significant improvement in medical student education.

We also investigated whether the rotation site-specificity impacted the students’ clerkship experience. Our results indicated that the students’ interest was significantly higher in the group who rotated through an inpatient and an outpatient site, however, this relation became insignificant after FDR correction. Our findings are consistent with the previous studies, which showed an improved experience with the ambulatory rotation [14].

As a future direction, additional interventions, such as resilience skills training program [15], the arts-based curriculum for neurology residents [16], and increasing resident involvement in the design and development of the neurology clerkship rotation [17] can be implemented to improve residents teaching skills and medical student experience and education.

Our study is limited by the lack of a comparator group and longitudinal follow-up of medical students’ career choices, identification of other factors that affected students’ experience, such as the impact of neurology faculty and objective measures of confidence in managing neurological conditions. Another limitation is that we have used an internally-generated four-point Likert scale (with no further tests for further internal consistency-see appendix) as a rating tool that has not been previously validated. Also, the residents did not complete any surveys, and there was no assessment of residents’ teaching skills before and after the workshop. Studies with previously validated measurement tools are needed to confirm our findings.

## Conclusion

In light of the current and anticipated shortage of neurologists, coupled with the increasing incidence of various neurologic conditions, developing additional future neurologists, is imperative. Efforts are underway to increase medical student interest in neurology and decrease neurophobia. We evaluated the impact neurology residents have on third-year medical students during their core neurology clerkship and, in fact, confirmed our hypothesis. Resident teaching has a positive impact on medical students’ perception and understanding of clinical neurology.

In this study, we demonstrated that through the effective teaching of neurology residents, there was an improvement in medical students’ neurologic educational experience and an increase in their interest in neurology**.** Empowering residents to take an active teaching role during the core neurology clerkship, after adequately equipping them with training, seems to be an effective method for improving medical students’ neurologic experience and education during the clerkship. This increased interest may have benefits at the societal level in terms of career choice. The role of residents in medical students’ neurology education is crucial and holds great promise, but it needs to be reconsidered at a national level to ensure proper high-quality standards on both the students’ and the residents’ side.

## Supplementary information

**Additional file 1.**

## Data Availability

The datasets analyzed during the current study available from the corresponding author on reasonable request.

## Abbreviations

MMS: McGovern Medical School
p-val: *p*-values
UTHealth: University of Texas Health Science Center at Houston
SD: Standard deviation
