# Supplementary material for: The role of residents in medical students’ neurology education: current status and future perspectives
Source: BMC Med Educ. 2020 Apr 16;20:115. doi: 10.1186/s12909-020-02036-1 (PMC7164350; doi:10.1186/s12909-020-02036-1)
Supplement: Supplementary file 1 — Additional file 1. [file 12909_2020_2036_MOESM1_ESM.docx]

**Supplement: The role of residents**

**in medical students’ neurology education:**

**current status and future perspectives**

Zafer Keser^1*^, MD, Yvo A Rodriguez^1^ MD, Jennifer Tremont^1^, BS, Peggy H Hsieh^2^, PhD, Louise D. McCullough, MD, PhD,^1^ Stefano Sandrone^3^, PhD, Erin F Stimming^1^, MD

**Pre-Rotation Survey**

How confident do you feel about managing neurological conditions?

1. Not confident at all
2. Not confident
3. Somewhat confident
4. Very confident

How interested are you in doing neurology residency?

1. Not interested at all.
2. Not interested.
3. Somewhat interested.
4. Extremely interested.

**Post-Rotation Survey**

During your rotation, do you feel that your resident(s) have adequate time to teach?

1. There was no teaching at all.
2. Not a lot of time was spent on teaching.
3. Somewhat yes, though time was limited.
4. Definitely yes, they took time to teach.

During your rotation, how effective were your residents as teachers?

1. Extremely ineffective teacher.
2. Not an effective teacher.
3. Somewhat effective teacher.
4. Extremely effective teacher.

How did your resident(s) affect your overall experience of neurology clerkship?

1. Affected my experience extremely negatively.
2. Affected my experience somewhat negatively.
3. Affected my experience somewhat positively.
4. Affected my experience extremely positively.

Was/Were your resident(s) good role models in terms of professionalism and bedside manner?

1. Definitely not.
2. Probably not.
3. Probably yes.
4. Definitely yes.

How valuable was the neurology rotation experience?

1. Not valuable at all.
2. Not valuable.
3. Slightly valuable.
4. Very Valuable.

How confident do you feel about managing neurological conditions?

1. Not confident at all
2. Not confident
3. Somewhat confident
4. Very confident

How interested are you in doing neurology residency?

1. Not interested at all.
2. Not interested.
3. Somewhat interested.
4. Extremely interested.
